# German evidence and consensus based guidelines 2010 for the treatment of juvenile idiopathic arthritis (JIA)

**DOI:** 10.1186/1546-0096-9-S1-P181

**Published:** 2011-09-14

**Authors:** G Dueckers, N Guellac, M Arbogast, G Dannecker, I Foeldvari, M Frosch, G Ganser, A Heiligenhaus, G Horneff, A Illhardt, I Kopp, R Krauspe, B Markus, H Michels, M Schneider, W Singendonk, H Sitter, M Spamer, N Wagner, T Niehues

**Affiliations:** 1 HELIOS Children’s Hospital, Krefeld, Germany; 2 German Federal Armed Forces central hospital, Koblenz, Germany; 3 Rheumazentrum Oberammergau, Oberammergau, Germany; 4 Olgahospital, Stuttgart, Germany; 5 Klinikum Eilbeck, Hamburg, Germany; 6 University Children’s Hospital, Muenster, Germany; 7 St. Josef Stift, Sendenhorst, Germany; 8 St. Franziskus Hospital, Münster, Germany; 9 Asklepios Children’s Hospital, St. Augustin, Germany; 10 Arbeitsgemeinschaft der Wissenschaftlichen Medizinischen Fachgesellschaften (AWMF), Marburg, Germany; 11 University Department of Orthopaedics, Duesseldorf, Germany; 12 Deutsche Rheuma Liga e.V., Bonn, Germany; 13 German Centre for Paediatric and Adolescent Rheumatology, Garmisch Partenkirchen, Germany; 14 University Department of Endocrinology, Diabetology and Rheumatology, Duesseldorf, Germany; 15 Berlin-Schoeneberg, Germany; 16 University Department of Surgical Research, Marburg, Germany; 17 University Children’s Hospital, Aachen, Germany

## Background

To improve the quality of care for patients with JIA, standardization of treatment is mandatory. We present a clinical practice guideline (CPG) for the treatment of JIA. It is based on the existing CPG of 2005^*^ and 2008^#^ (^*^published as book chapter; ^#^peer reviewed publication: Clinical Research and Practice in Paediatrics 2008; 220: 392 - 402).

## Methods

We performed a systematic literature analysis in PubMed (deadline: 15^th^ January 2010, terms "juvenile idiopathic (rheumatoid) arthritis", "therapy", limits: “humans”, “published in the last 3 years”, “all child 0-18 years”, “clinical trial”) and evaluated relevant studies for quality of methodology. A formal consensus process, i.e. Nominal group technique and Delphi method, was conducted at three moderated consensus conferences at Düsseldorf or Krefeld (Germany) on May 9^th^, 2007, August 1^st^, 2007 and January 15^th^, 2010. Conferences were attended by 95% of the representatives who had been nominated by their scientific society or organizations.

## Results

15 consensus statements and key notes regarding drug therapy, symptomatic and surgical management of JIA were compiled and judged strictly by the criteria of Evidence-Based Medicine (presented on the poster).

## Conclusions

Currently, we recommend that JIA is treated with NSAR followed by local GC and/ or MTX as first line treatment. Other interventions e. g. the role of biologic agents, physiotherapy, and arthroscopy are discussed strictly on the basis of literature available. Our CPG will require a frequent update, as the number of randomized trials is increasing and the new potential drugs will become available.

